# Sedentary Behavior, Obesity, and Disabilities in Community-Dwelling Older Adults: Analysis of the Brazilian National Health Survey 2019

**DOI:** 10.3390/healthcare12161625

**Published:** 2024-08-15

**Authors:** Bruno Prates Freitas, Letícia Martins Cândido, Katia Jakovljevic Pudla Wagner, Ana Cristina Rodrigues Lacerda, Vanessa Amaral Mendonça, Roberta De Micheli, Alessandro Sartorio, Núbia Carelli Pereira de Avelar, Ana Lúcia Danielewicz

**Affiliations:** 1 Department of Health Sciences, Laboratory of Aging, Resources and Rheumatology, Universidade Federal de Santa Catarina, Araranguá 88906-072, SC, Brazilnubia.carelli@ufsc.br (N.C.P.d.A.); 2 Postgraduate Program in Public Health, Universidade Federal de Santa Catarina, Florianópolis 88040-900, SC, Brazil; leticia.candido96@gmail.com; 3 Coordination of Biosciences and Unified Health, Universidade Federal de Santa Catarina, Curitibanos 89520-000, SC, Brazil; katia.wagner@ufsc.br; 4 Departament of Physiotherapy, Universidade Federal dos Vales do Jequitinhonha e Mucuri, Diamantina 39100-000, MG, Brazil; lacerdaacr@gmail.com (A.C.R.L.);; 5 Istituto Auxologico Italiano, IRCCS, Experimental Laboratory for Auxo-Endocrinological Research, 28824 Piancavallo-Verbania, Italy

**Keywords:** activities of daily living, ageing, obesity, sedentary behavior

## Abstract

Research suggests that sedentary behavior (SB) and obesity are associated with disabilities in basic activities (BADL) and instrumental (IADL) activities of daily living. However, there is a lack of studies investigating this association in community-dwelling older adults. Thus, the aim of this study was to investigate the association between different SB typologies, isolated and in conjunction with obesity, and their associations with BADL and IADL disabilities in community-dwelling Brazilian older adults. This was a cross-sectional study using data from older adults (≥60 years) who participated in the Brazilian National Health Survey (2019). The exposures were obesity (BMI > 27 kg/m^2^) and the amount of time spent daily on SB watching television (SB TV < 3 and ≥3 h/day) and engaging in leisure activities (SB leisure < 3 and ≥3 h/day), analyzed both separately and jointly. The outcomes were BADL and IADL disabilities. The main results showed that isolated SB TV ≥ 3 h/day (OR: 1.26; 95% CI: 1.14; 1.39) and SB TV ≥ 3 h/day combined with obesity (OR: 1.55; 95% CI: 1.37; 1.75) increased the odds of BADL and IADL disabilities. Obesity alone (OR: 1.21; 95% CI: 1.07; 1.36) increased only the odds of BADL disabilities. Moreover, SB leisure ≥ 3 h/day without obesity reduced the odds of IADL disabilities (OR: 0.56; 95% CI: 0.41; 0.76). Ideally, older adults should be encouraged to prevent obesity, reduce excessive periods spent in SB watching TV, and increase the daily periods spent in leisure activities, thus minimizing the likelihood of disabilities in functional activities.

## 1. Introduction

According to estimates from the World Health Organization, by 2050 the world population aged 60 and over is projected to be twice as large as it is currently and, by the same date, 80% of the world’s older people will live in low to middle-income countries [1]. This aging population phenomenon highlights the importance of addressing common issues faced by older adults, such as sedentary lifestyle, obesity and disabilities [2,3,4].

Sedentary behavior (SB) is characterized as any waking activity with low energy expenditure (≤1.5 metabolic equivalents) while the individual stands in a sitting, reclining or lying posture [5]. It can be performed in different activities, such as the periods spent at screens (e.g., televisions, computers, or tablets), reading, eating, socializing, working, and driving vehicles [6]. Silveira et al. (2022) estimated that 31% of the adults and older adults (≥18 years old) in different continents (Africa, America, Europe, and Oceania) spent more than 8 h per day in at least one of these types of SB [2]. In older adults, SB in front of television for more than 3 h per day requires attention because it is associated with different morbidities, including diabetes mellitus, hypertension, depression, as well as heart, respiratory, joint, and musculoskeletal diseases [7]. Furthermore, it has been shown that SB is closely associated with obesity [8].

The prevalence of obesity in the older populations was estimated to be around 19.0% in Europe, while in the United States was 37.5% in men and 39.4% in women over 60 years old in 2014 [9]. In Brazil, 17.9% of those aged 65 to 74 years and 15.8% of those aged 75 years or older were obese in 2019 [3]. Obesity is a relevant and growing public health problem in the world as it is associated with various chronic diseases and disabilities [10].

Disabilities can be measured through difficulties in performing basic activities of daily living (BADL), such as sitting, standing up, and walking from one room to another, and instrumental activities of daily living (IADL), which include using transportation, shopping, and managing finances [4]. Both conditions, BADL and IADL disabilities, are highly prevalent and affect 17.6% and 46.3% of the Brazilian older people, respectively [11]. The ability to independently perform BADL and IADL can be impaired by excessive periods in SB, which leads to a decrease in time spent on active behaviors, as well as induces poor dietary quality [12]. In addition, obesity can be an aggravating factor in this association due to the increased load on joint structures and chronic inflammatory state associated with adipose tissue accumulation, factors that lead to physical performance impairments [13].

Gomes et al. (2021) performed a systematic review that analyzed studies with samples of community-dwelling people over 60 years old living in America, Europe, Asia and Australia and investigated the relationship between different forms of assessing the SB (e.g., number of steps per day, metabolic equivalents, and moderate and vigorous physical activity) with the presence of disabilities; in all longitudinal and most cross-sectional studies, the authors found that SB was associated with BADL and IADL disabilities [14]. However, most studies investigated the association between SB and disabilities in samples of individuals with specific clinical conditions such as fractures, osteoporosis, and joint symptoms [15,16,17]. Given this context, the present study innovates by demonstrating two typologies of SB, both isolated and together with obesity, and their association with disabilities in a sample of Brazilian older adults without specific comorbidities.

We believe that knowledge about different types of SB and the recommended daily periods that may contribute to the occurrence of disabilities in older individuals is extremely relevant. This enables health professionals to offer more specific and targeted actions and guidance on disability prevention for this population. Therefore, this study aimed to investigate the association between different SB typologies, isolated and jointly with obesity, and its associations with BADL and IADL disabilities in community-dwelling Brazilian older adults.

## 2. Methods

### 2.1. Study Design and Population

A cross-sectional study with secondary data from the 2019 Brazilian National Health Survey and exempt from the Institutional Review Board (IRB) assessment. The data were collected by the Brazilian Institute of Geography and Statistics (IBGE) and approved by the National Research Ethics Committee of the National Health Council, under number 3.529.376, issued in 23 August 2019. Written informed consent was signed by all participants. The analysis focused exclusively on data collected from the sampled population aged 60 years and older, who do not represent the entire sample of the 2019 NHS [18].

### 2.2. Sampling and Data Collection

Sampling was formulated by clusters in three stages. In the first, the primary sampling units were stratified based on a pre-established set of sections of the national territory used for studies by the IBGE Integrated Household Survey System—the master sample. In the second stage, a fixed number of households registered in the National Register of Addresses for Statistical Purposes were selected in each primary sampling unit by simple random sampling. The third stage consisted of randomly choosing a resident aged 15 or older in each household to answer the survey questionnaire. The interviews were scheduled according to the participant’s convenience, and two or more visits were scheduled for each household. The inclusion criterion for this study was only individuals aged 60 or older, without any specific chronic diseases, who had answered all questionnaires and/or variables of interest. More information about the sampling process and data collection is provided in a previous study [18,19].

### 2.3. Independent Variables

The SB was evaluated through a questionnaire constructed by technicians from the Brazilian Ministry of Health based on validated instruments [20], which included the following questions: (1) “On average, how many hours a day do you usually watch television (TV)” and (2) “In a day, how many hours of your free time (excluding work) do you usually use the computer, tablet, or mobile phone for leisure in activities such as using social networks, watching the news, videos, playing games, etc. The response options for each one of the two types of SB (SB TV and SB leisure) were dichotomized: (1) <3 h/day and ≥3 h/day considering the previous study [7].

Obesity was evaluated during the interviews at the participant’s homes, using electronic scales and portable stadiometers to measure weight and height, respectively. The body mass index (BMI), defined as the ratio between weight in kilograms and the square of height in meters, was used to classify obesity considering values > 27 kg/m^2^ [21]. Then, SB TV and SB leisure were classified together with the presence of obesity as follows:

Typology A: (1) SB TV < 3 h/day and no obesity; (2) only SB TV ≥ 3 h/day; (3) only obesity, and (4) SB TV ≥ 3 h/day + obesity.

Typology B: (1) SB leisure < 3 h/day and no obesity; (2) only SB leisure ≥ 3 h/day; (3) only obesity, and (4) SB leisure ≥ 3 h/day + obesity.

### 2.4. Dependent Variables

The outcomes were assessed during the interviews through a self-reported questionnaire with questions that analyzed the presence of BADL and IADL disabilities, considering the positive answers for minor/major difficulty or inability to perform at least one of seven BADL: eating; bathing, using the bathroom, dressing, walking from one room to another, getting up or laying down from bed, and sitting or getting up from a chair, and at least one of five IADL: shopping, managing finances, taking medication, going to the doctor, and using means of transportation. The outcomes categorization was based on our previous study [22].

### 2.5. Adjustment Variables

The following adjustment variables were adopted: binary concept of sex (male and female), age group (60–69, 70–79, and 80 years or over), educational level (no formal education, 1–4, 5–8, 9–11 years, and 12 years or more), marital status (married, divorced/single, and widowed), self-perceived health (very good, good, regular, poor, and very poor), the number of self-reported chronic diseases (0, 1 or ≥2), self-reported depressive symptoms (yes or no), and level of leisure-time physical activity (insufficiently active with less than 150 min/week of physical activity and sufficiently active with over 150 min/week of physical activity). To define leisure-time physical activity, self-reports of light/moderate physical activity (e.g., walking, weight training, and water gymnastics) and vigorous physical activity (e.g., running, soccer, basketball, tennis, and aerobics) were used. More information about the classification is described elsewhere [23].

### 2.6. Data Analysis

Statistical analyses were performed using the Stata software (version 14.0) (Stata Corp., College Station, TX, USA). Descriptive analyses were performed for all variables, calculating the prevalence and respective 95% confidence intervals (CI 95%). To test the associations between independent and dependent variables, multivariable logistic regression analysis was used and considered statistically significant when *p* < 0.05 was obtained from the estimate of the odds ratio (OR) unadjusted and adjusted and the respective CI 95%. All analyses considered the effect of study design, incorporating sample weights using the ‘svy’ command.

## 3. Results

This study analyzed 22,728 older adults, who were predominantly females (55.3%), married (44.3%), aged between 60 and 69 years (55.4%), with 1–4 years of formal education (61.1%), and with a per capita household income less than one minimum wage (Brazilian real) (41.3%). Most of them did not report depressive symptoms (79.3%), related very good/good health (46.9%), were insufficiently active (80.6%), and over half of the sample reported two or more chronic diseases (50.5%) (Table 1).

The prevalence of BADL disabilities was 19.9% (95% CI: 19.3; 20.5) and of IADL disabilities was 31.8% (95% CI: 31.2; 32.5). Regarding the exposures analyzed, 41.5% (95% CI: 40.8; 42.2) of the participants were obese, 28.8% (95% CI: 28.2; 29.5) related SB TV ≥ 3 h/day, and 5.2% (95% CI: 4.8; 5.5) related SB leisure ≥ 3 h/day. The prevalence of SB TV ≥ 3 h/day + obesity and SB leisure ≥ 3 h/day + obesity was 13.2% (95% CI: 12.7; 13.7) and 2.6% (95% CI: 2.4; 2.9), respectively.

**Figure 1:**
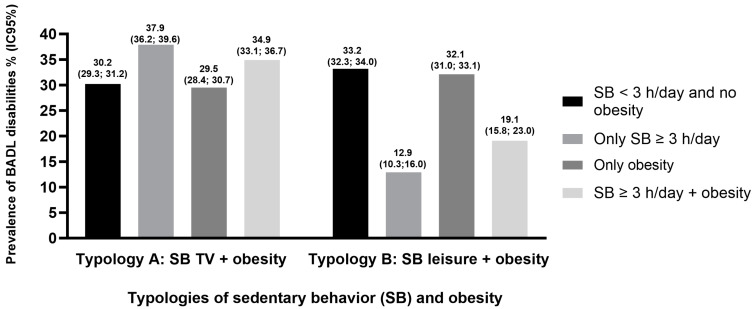
Prevalence of Basic Activities of Daily Living (BADL) disabilities according to typologies of sedentary behavior and obesity in older adults. National Health Survey, Brazil, 2019.

**Figure 2:**
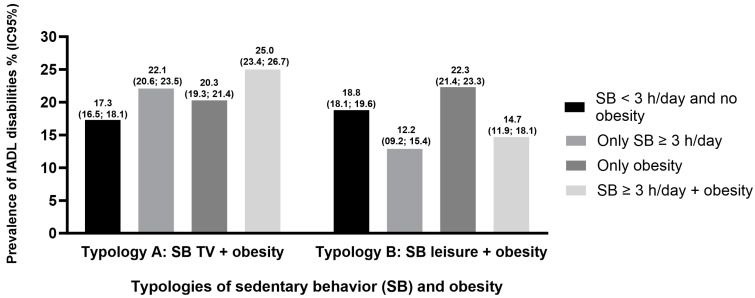
Prevalence of Instrumental Activities of Daily Living (IADL) disabilities according to typologies of sedentary behavior and obesity in older adults. National Health Survey, Brazil, 2019.

Figure 1 and Figure 2 illustrate the prevalence of BADL and IADL disabilities according to typologies of SB and obesity, respectively. The prevalence of BADL disabilities for SB TV ≥ 3 h/day + obesity was 34.9% (95% CI: 33.1; 36.7) and for SB leisure ≥ 3 h/day + obesity was 19.1 (95% CI: 15.8; 23.0). The prevalence of IADL disabilities for SB TV ≥ 3 h/day + obesity was 25.0% (95% CI: 23.4; 26.7) and for SB leisure ≥ 3 h/day + obesity was 14.7% (95% CI: 11.9; 18.1).

Considering the adjusted analyses, older adults with isolated SB TV ≥ 3 h/day, isolated obesity, and with SB TV ≥ 3 h/day + obesity showed 1.21 (95% CI: 1.07; 1.36), 1.26 (95% CI: 1.14; 1.39), and 1.55 (95% CI: 1.37; 1.75) higher odds of BADL disabilities, respectively, compared to those non obese that spent < 3 h/day in SB TV. The odds of IADL disabilities were significantly higher for the older adults with isolated SB TV ≥ 3 h/day (OR: 1.38; 95% CI: 1.24; 1.54) and SB TV ≥ 3 h/day + obesity (OR: 1.25; 95% CI: 1.12; 1.40) compared to those non obese with SB TV < 3 h/day (Table 2).

Moreover, older adults with isolated obesity had 1.28 (95% CI: 1.18; 1.39) more odds of BADL disabilities compared to those with SB leisure < 3 h/day without obesity. Nevertheless, the participants with isolated SB leisure ≥ 3 h/day were 44% (OR: 0.56; 95% CI: 0.41; 0.76) less likely to reported IADL disabilities compared to those in SB leisure < 3 h/day without obesity (Table 2).

## 4. Discussion

The main results showed that SB TV (≥3 h/day), isolated and jointly with obesity, was associated with higher odds of both BADL and IADL disabilities. Isolated obesity was associated only with higher odds of BADL disabilities, while isolated SB leisure (≥3 h/day) was associated with lower odds of IADL disabilities. Furthermore, the high prevalence of BADL disabilities (37.9%) was observed in non-obese older adults with ≥3 h/day of SB TV, while IADL disabilities (25.0%) were higher in obese older adults with SB TV ≥ 3 h/day.

Studies describing prevalence rates of disabilities according to SB in older adults are still scarce in Brazil. In a previous study published by our research group [7], the prevalence of musculoskeletal conditions that potentially lead to disabilities was higher in non-obese older individuals who spent ≥ 3 h/day in SB TV (around 40%), consistent with the present findings. In another Brazilian study conducted with non-obese older adults, the association between SB total and BADL and IADL disabilities was also observed, and although the authors did not describe the prevalence rates, prolonged hours in SB increased significantly the odds of both outcomes [24].

In our results, obesity was associated with BADL disabilities when analyzed alone and jointly with SB TV. These findings are consistent with other evidence pointing to the relationship between obesity and BADL disabilities in older adults [12,25]. This effect is probably due to the nature of BADL, which depends more intrinsically on the individual’s ability to move and lift their weight [25,26]. High body mass can cause osteoarticular disorders that lead to greater difficulties in performing movements included in the BADL spectrum, such as sitting/standing up from a chair and walking from one room to another [12]. The relationship between obesity and impaired mobility has already been presented by Ramírez-Vélez et al. (2019), who analyzed data from 20,507 Colombian community-dwelling older adults and concluded that obesity was associated with decreased walking speed and, consequently, BADL disabilities [27]. In addition, obesity is known to be a chronic inflammatory disease associated with the development of various musculoskeletal morbidities, through mechanisms that include oxidative stress, systemic inflammation via adipose tissue-derived cytokines (e.g., TNF-α, IL-6), and mechanical strain on weight-bearing joints. These factors accelerate cartilage wear, contribute to muscle mass loss, and disrupt bone metabolism, leading to reduced bone quality and increased risk of functional impairments [28].

High odds of BADL disabilities were found in obese older adults who spent ≥ 3 h per day on SB TV, and the presence of obesity increased the magnitude of this association compared with non-obese who spent less time on SB TV. The relationship between SB TV and BADL disabilities can be explained mainly by the behaviors associated with watching television for long periods [29,30]. Like other passive sedentary activities, watching television stimulates social isolation, leading to the decline in executive functions essential for daily living activities [29,30,31]. In addition, SB in passive contexts, including watching television, is linked to increased risks of cardiovascular disease, type 2 diabetes mellitus, and other negative health outcomes [6]. Depending on the severity level, the symptoms of such morbidities can limit the performance of activities requiring greater physical effort, consequently leading to BADL disabilities [4]. Other mechanisms can also explain the correlation between SB and BADL disabilities, including reduced muscle strength and flexibility due to prolonged sitting, decreased stamina from poor circulation, and cognitive decline affecting decision-making skills necessary for daily activities [14]. Additionally, a Brazilian study identified a positive correlation between high SB levels and BADL disabilities mediated by factors such as aerobic resistance and lower limb flexibility [4].

Our results showed that SB leisure ≥ 3 h/day reduced the odds of IADL disabilities. The daily periods spent in SB using devices such as tablets and/or computers usually involve more active cognitive activities than watching television, requiring concentration and reading [32]. In addition, unlike the habit of watching television, SB leisure subsidizes interpersonal interaction, which prevents the deterioration of the mental health of older people [30]. Moreover, it seems that older adults who perform SB leisure are more likely to perform physical activities. A study that analyzed 1580 Japanese older adults showed an association between SB TV with less physical activity of moderate to vigorous intensity, while more intellectually active practices such as reading, using the computer, and the internet were associated with greater physical activity of the same intensity [16]. Furthermore, it is possible that SB leisure might specifically act as a protective factor for IADL disabilities, requiring a more cognitive involvement which is necessary for carrying out these activities [32].

None of the analyses indicated that isolated obesity was associated with greater odds of IADL disabilities. In the literature, the relationship between obesity and type of disability is still inconsistent [33,34]. While some studies have only found a relationship between obesity and disabilities in BADL, others have pointed to obesity as a risk factor for both; nevertheless, the relationship between obesity and BADL disabilities appears more frequently [20,27,29,35]. It is important to highlight sampling and methodological differences between studies, such as the tools used to measure obesity (e.g., BMI or waist circumference) and study design [36]. A longitudinal study analyzed 1040 older adult Brazilians using waist circumference to measure obesity and found an association between abdominal obesity and IADL disabilities, although BADL was not investigated [36]. It is clear, therefore, that the association between obesity and disabilities deserves further investigation, preferably via standardized analysis methods.

Despite the pertinent findings of this study, it is important to emphasize a considerable limitation, which was the use of self-reported and under or overestimation of the inquired measures. Furthermore, the cross-sectional design does not support causality between the dependent and independent variables investigated [37]. Still, some of the findings are difficult to compare with the literature due to the lack of standardization of the analyzed variables—SB typologies [11], obesity measurements [26], and tools of disabilities evaluation [20]—which may alter the final results of each study.

As a strong point, it is worth highlighting the originality of our study, which explores the gap in the literature on the relationship between different types of SB, obesity, and disabilities, in addition to the national coverage of the sample. Ideally, older adults should be encouraged to prevent obesity, reduce the excessive time spent in front of television, as well as to increase the daily time spent in leisure activities, thus minimizing the likelihood of disabilities in functional activities. Furthermore, the fight against obesity can prevent disability among older adults, in addition to the various other negative outcomes already described in the literature. Lastly, future Brazilian studies should investigate the association between SB typologies and disabilities, considering also different durations of SB.

In conclusion, different associations between SB typologies and obesity with disabilities were observed in our sample of Brazilian community-dwelling older adults. Isolated SB TV ≥ 3 h/day and jointly with obesity increased the odds of both, BADL and IADL disabilities, whereas isolated obesity increased only the odds of BADL disabilities. On the other hand, isolated SB leisure ≥ 3 h/day decreased the odds of IADL disabilities.

## Figures and Tables

**Table 1:** Description of sociodemographic, behavioral, and health condition variables according to the total sample and for disabilities in basic (BADL) and instrumental (IADL) activities of daily living in older adults. National Health Survey, Brazil, 2019.

Variables	Total	BADL Disabilities	IADL Disabilities
	% (95% CI)	% (95% CI)	% (95% CI)
Sex [n = 22,728]			
Male	44.6 (43.9; 45.3)	16.6 (15.8; 17.4)	24.7 (23.8; 25.6)
Female	55.3 (54.6; 56.0)	22.6 (21.8; 23.3)	37.6 (36.7; 38.5)
Age range (years) [n = 22,728]			
60–69	55.4 (54.7; 56.1)	14.1 (13.4; 14.7)	20.2 (19.4; 20.9)
70–79	31.3 (30.7; 32.0)	21.9 (20.9; 22.9)	37.8 (36.6; 39.0)
≥80	13.1 (12.6; 13.6)	39.8 (38.0; 41.6)	66.8 (65.0; 68.6)
Years of formal education [n = 21,869]			
No education	21.9 (21.3; 22.6)	28.2 (27.0; 29.5)	51.7 (50.2; 53.2)
1–4	37.4 (36.7; 38.2)	21.6 (20.6; 22.5)	35.3 (34.2; 36.4)
5–8	12.8 (12.3; 13.3)	18.6 (17.0; 20.2)	24.9 (23.2; 26.6)
9–11	16.9 (16.3; 17.4)	14.1 (13.0; 15.4)	19.5 (18.2; 20.9)
≥12	10.7 (10.2; 11.2)	10.9 (9.6; 12.4)	13.2 (11.8; 14.8)
Per capita household income (minimum wage) [n = 22,725]			
<1	41.3 (40.5; 42.0)	24.0 (23.1; 24.9)	40.3 (39.3; 41.4)
≥1 and <2	30.1 (29.4; 30.8)	19.7 (18.7; 20.8)	31.1 (29.9; 32.3)
≥2	28.5 (27.8; 29.3)	14.1 (13.2; 15.1)	20.4 (19.3; 21.5)
Marital status [n = 22,728]			
Married	44.3 (43.5; 45.0)	17.1 (16.3; 17.9)	26.6 (25.6; 27.5)
Divorced/single	28.7 (28.1; 29.3)	18.3 (17.3; 19.3)	26.8 (25.7; 27.9)
Widower	26.9 (26.3; 27.5)	26.3 (25.1; 27.5)	45.9 (44.5; 47.3)
Depressive symptoms [n = 22,728]			
No	90.0 (89.5; 90.4)	16.8 (16.2; 17.4)	28.4 (27.7; 29.0)
Yes	9.9 (9.5; 10.4)	48.0 (45.8; 50.2)	63.2 (61.1; 65.3)
Self-rated health [n = 22,728]			
Very good/good	46.9 (46.2; 47.6)	9.1 (8.5; 9.7)	17.1 (16.3; 17.9)
Regular	41.8 (41.1; 42.5)	23.3 (22.4; 24.3)	38.9 (37.8; 39.9)
Bad/very bad	11.2 (10.8; 11.6)	52.4 (50.4; 54.4)	67.5 (65.6; 69.3)
Physical activity at leisure [n = 22,585]			
Insufficiently active	80.6 (80.0; 81.2)	22.6 (22.0; 23.3)	36.0 (35.3; 36.8)
Sufficiently active	19.3 (18.7; 19.9)	8.8 (7.9; 9.8)	14.8 (13.7; 15.9)
Number of chronic diseases [n = 21,725]			
0	19.9 (19.3; 20.4)	10.5 (9.6; 11.5)	19.0 (17.8; 20.3)
1	29.5 (28.8; 30.2)	14.5 (13.6; 15.4)	26.3 (25.1; 27.4)
≥2	50.5 (49.8; 51.2)	26.9 (26.0; 27.9)	40.5 (39.5; 41.5)
N total (not weighted)	22,728		

95% CI: 95% confidence interval.

**Table 2:** Multivariate logistic regression between sedentary behavior (SB) typologies, obesity, and disabilities in basic (BADL) and instrumental (IADL) activities of daily living in older adults. National Health Survey, Brazil, 2019.

Exposures	BADL Disabilities		IADL Disabilities	
	Crude	Adjusted *	Crude	Adjusted *
	OR (95% CI)	OR (95% CI)	OR (95% CI)	OR (95% CI)
Typology A				
SB TV < 3 h/day and no obesity	Ref.	Ref.	Ref.	Ref.
SB TV ≥ 3 h/day and no obesity	**1.35 (1.22; 1.49)**	**1.21 (1.07; 1.36)**	**1.40 (1.29; 1.52)**	**1.38 (1.24; 1.54)**
Only obesity	**1.26 (1.12; 1.32)**	**1.26 (1.14; 1.39)**	0.96 (0.89; 1.03)	1.02 (0.93; 1.11)
SB TV ≥ 3 h/day + obesity	**1.59 (1.43; 1.77)**	**1.55 (1.37; 1.75)**	**1.23 (1.12; 1.35)**	**1.25 (1.12; 1.40)**
Typology B				
SB leisure < 3 h/day and no obesity	Ref.	Ref.	Ref.	Ref.
SB leisure ≥ 3 h/day and no obesity	**0.58 (0.45; 0.78)**	1.01 (0.74; 1.40)	**0.29 (0.23; 0.38)**	**0.56 (0.41; 0.76)**
Only obesity	**1.23 (1.15; 1.32)**	**1.28 (1.18; 1.39)**	0.95 (0.89; 1.01)	0.98 (0.91; 1.06)
SB leisure ≥ 3 h/day + obesity	**0.74 (0.57; 0.95)**	1.12 (0.84; 1.50)	**0.47 (0.38; 0.59)**	0.88 (0.67; 1.15)

In bold: statistically significant; * = Adjusted for sex, age group, years of formal education, per capita household income regarding minimum wages, marital status, depressive symptoms, self-rated health, number of chronic diseases, and level of leisure-time physical activity.

## Data Availability

The data that support the findings of this study are available on request from the corresponding author due to restrictions—ethical reasons.
